# ADAMTS proteases in cardiovascular physiology and disease

**DOI:** 10.1098/rsob.200333

**Published:** 2020-12-23

**Authors:** Salvatore Santamaria, Rens de Groot

**Affiliations:** 1 Centre for Haematology, Imperial College London, Du Cane Road, London W12 0NN, UK; 2 Institute of Cardiovascular Science, University College London, 51 Chenies Mews, London WC1E 6HX, UK

**Keywords:** ADAMTS, proteoglycans, atherosclerosis, aortic aneurysms, heart valve, cardiovascular

## Abstract

The a disintegrin-like and metalloproteinase with thrombospondin motif (ADAMTS) family comprises 19 proteases that regulate the structure and function of extracellular proteins in the extracellular matrix and blood. The best characterized cardiovascular role is that of ADAMTS-13 in blood. Moderately low ADAMTS-13 levels increase the risk of ischeamic stroke and very low levels (less than 10%) can cause thrombotic thrombocytopenic purpura (TTP). Recombinant ADAMTS-13 is currently in clinical trials for treatment of TTP. Recently, new cardiovascular roles for ADAMTS proteases have been discovered. Several ADAMTS family members are important in the development of blood vessels and the heart, especially the valves. A number of studies have also investigated the potential role of ADAMTS-1, -4 and -5 in cardiovascular disease. They cleave proteoglycans such as versican, which represent major structural components of the arteries. ADAMTS-7 and -8 are attracting considerable interest owing to their implication in atherosclerosis and pulmonary arterial hypertension, respectively. Mutations in the *ADAMTS19* gene cause progressive heart valve disease and missense variants in *ADAMTS6* are associated with cardiac conduction. In this review, we discuss in detail the evidence for these and other cardiovascular roles of ADAMTS family members, their proteolytic substrates and the potential molecular mechanisms involved.

## 1. Introduction

**Figure 1.**
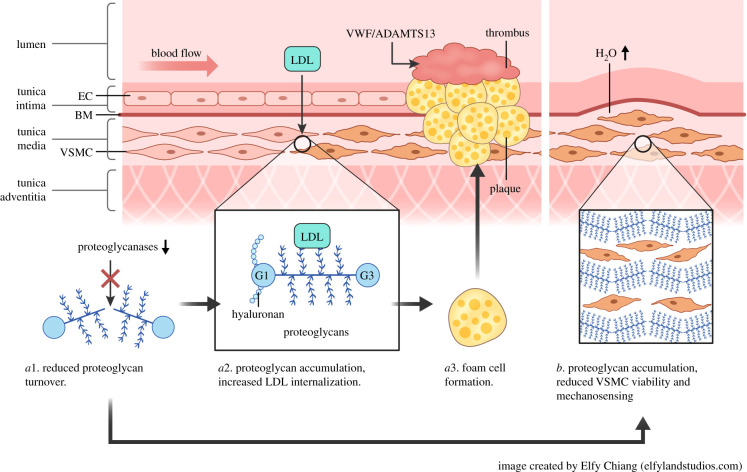
Involvement of ADAMTS proteases in cardiovascular physiology and disease. Atherosclerosis and haemostasis: decreased proteoglycanase activity (*a*1) is associated with proteoglycan accumulation, increased low-density lipoprotein (LDL) internalization (*a*2) and foam cell formation (*a*3), which eventually leads to formation of an atherosclerotic plaque. A thrombus can form on top of a plaque if collagen is exposed to the blood. ADAMTS-13 regulates the capacity of von Willebrand Factor (VWF) to recruit platelets to the site of collagen exposure and initiate thrombus formation. In the aorta, reduced proteoglycanase activity (*a*1) causes proteoglycan accumulation, increased osmotic pressure, reduced viability of vascular smooth muscle cells (VSMC) and disruption of mechanosensing (*b*). BM, basal membrane; EC, endothelial cell; VWF, von Willebrand factor.

**Figure 2.**
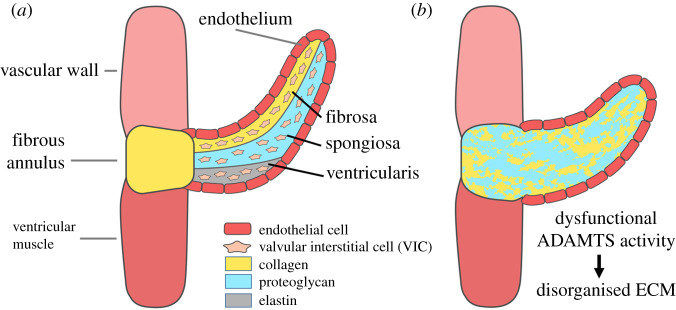
Extracellular matrix (ECM) organization in cardiac valves. (*a*) The ECM composition in a mature valve is shown. The formation of the ventricularis and the fibrosa during development is a complex and tightly regulated molecular process in which ADAMTS proteases play an essential role. (*b*) Dysfunctional ADAMTS activity can lead to a certain degree of disorganized ECM, altered valve shape and leakage of the valve. Disorganized ECM can present as an abundance of proteoglycans, which can be owing to insufficient proteoglycanase activity or possibly owing to incorrect assembly of the ECM in the ventricularis (e.g. fibrillin-1 microfibrils/elastin). ADAMTS-1, -5 and -9 have been implicated in cardiac valve development by mouse studies and ADAMTS-19 by a human genetic disease.

The extracellular matrix (ECM) guides the formation of cardiovascular tissues during embryogenesis and supports it throughout adulthood by providing structural support, guidance of cell behaviour and sequestering of growth factors. In large arteries and blood vessels, collagen and elastic fibres provide essential structural support to prevent rupture. A healthy artery consists of three layers (tunicae): tunica adventitia, media and intima (figure 1). The intima is in contact with the vessel lumen and consists of endothelial cells (ECs) attached to a basal membrane rich in collagen IV, laminin, nidogen and heparan sulfate (HS) proteoglycans (PGs) (syndecan, perlecan). The tunica media is made up of vascular smooth muscle cells (VSMC), which express chondroitin-sulfate (CS)/dermatan-sulfate (DS)-PGs (CSPGs) (such a versican and aggrecan) and elastin. There are fenestrated sheets of elastin called lamellae between which there are collagen fibres, thin layers of PG-rich ECM and VSMCs. Elastin, which is distensible and has a low tensile strength, functions as an elastic reservoir and distributes stress evenly throughout the wall and onto collagen fibres [1]. The adventitia is the outermost layer of a blood vessel and consists of a collagen-rich ECM secreted by fibroblasts that prevents vascular rupture at very high pressures. The adventitia is also a reservoir of stem cells and plays an essential role in regulating the functions of cell populations in the tunica intima and media [2]. Various PGs are present in the different layers. In the tunica media, they contribute to the viscoelastic properties of the vessel wall. This function of PGs is mediated by their glycosaminoglycan (GAG) chains. Owing to their high negative charge, these GAGs attract counter-ions and water into the tissue [3,4]. Moreover, CSPGs such as versican and aggrecan are essential components of cardiac valve leaflets and, in particular, the middle ECM layer, the spongiosa, while elastin and collagens predominate in the ventricularis/atrialis and fibrosa layers, respectively [5,6] (figure 2). The ratios of PGs and the interspersed collagens/elastic fibres provide a means of balance between stiffness and flexibility of the cardiovascular tissues [7]. For this reason, remodelling of the vascular ECM by proteases secreted by both ECs and VSMCs is crucial to establish the mechanical properties of these tissues. Moreover, ECM degradation is required for migration and proliferation of VSMCs, as well as infiltration of inflammatory cells under pathological conditions. Among the proteases involved in this dynamic action, members of the matrix metalloproteinases (MMP) family have been extensively investigated owing to their ability to cleave the elastic ECM components such as collagens (MMP-1, -8, -13, -14) and elastin (MMP-12). In addition, proteases of the related family of a disintegrin and metalloproteinases (ADAMs) exert a fundamental role in the vascular ECM owing to their ability to selectively cleave the ectodomain of membrane proteins (shedding). The role of these metalloproteinase families in cardiovascular disorders has been exhaustively reviewed elsewhere [8,9].

This review focuses on a closely related metalloprotease family, the a disintegrin-like and metalloproteinase with thrombospondin motif (ADAMTS) and their (patho)physiological role in heart and blood vessels. In humans, the ADAMTS family comprises 19 secreted metalloproteinases as well as 7 ADAMTS-like proteins devoid of catalytic activity [10]. They share a common domain composition consisting of a signal peptide, a prodomain, a metalloproteinase catalytic domain (Mp, absent in the ADAMTS-like proteins), followed by non-catalytic ancillary domains such as a disintegrin-like (Dis) domain, a central thrombospondin-type I motif (TSR), a cysteine-rich (CR) domain, a spacer (Sp) domain, and, with the exception of ADAMTS-4, a various number of TSRs at the C-terminus (table 1). Some members have additional C-terminal domains, such as a mucin domain (present in ADAMTS-7 and ADAMTS-12), a Gon-1 like domain (in ADAMTS-20 and ADAMTS-9) and a PLAC (protease and lacunin) domain (in ADAMTS-2, -3, -6, -10, -12, 14, -16, -17, -18 and -19). Moreover, ADAMTS-13 is unique among ADAMTS proteases since it presents two CUB [complement subcomponent C1r/C1s/embryonic sea urchin protein Uegf (urchin epidermal growth factor)/bone morphogenic protein 1] domains [11,12]. A common feature of the family is the presence of a zinc-binding motif in the Mp domain, containing the consensus sequence HEXXHXXGXXH, in which the three underlined histidine residues coordinate a zinc atom, which together with a glutamate residue, exerts a catalytic role. This motif is followed C-terminally by a methionine residue that constitutes a structural turn (Met-turn) conserved within the metzincin family of metallopeptidases (comprising MMPs, ADAMs, ADAMTSs and astacins) [13].

**Table 1.** Summary of the cardiovascular roles, proteolytic functions and domain organization of the ADAMTS family. For the cardiovascular roles and diseases, the numbers in brackets indicate specific ADAMTS family members. CAD, coronary artery disease; PAH, pulmonary arterial hypertension; TAAD, thoracic aortic aneurysms and dissections; TTP, thrombotic thrombocytopenic purpura; VSMC, vascular smooth muscle cells; VWF, von Willebrand factor; WMS, Weill-Marchesani syndrome. The ADAMTS protein domains are abbreviated as follows: S, signal peptide; Pro, prodomain; Mp, metalloproteinase domain; Dis, disintegrin-like domain; CR, cysteine-rich domain; Sp, spacer domain; Muc, mucin-like domain; CUB, complement subcomponent C1r/C1s/embryonic sea urchin protein Uegf (urchin epidermal growth factor)/bone morphogenic protein 1; PL, protease and lacunin domain; GON, gon-1 like domain.

proteolytic function	family members	domain organisation	cardiovascular role	disease
proteoglycanases	ADAMTS-1/15	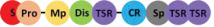	regulation of PG turnover, LDL internalisation **(5)**, development of cardiovascular system (**1, 5, 9)**	atherosclerosis **(4)** TAAD **(1, 4, 5)** PAH **(8)**
	ADAMTS-4	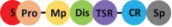		
	ADAMTS-5/8	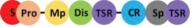		
	ADAMTS-9/20	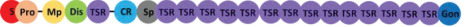		
procollagen N-propeptidases	ADAMTS-2/3/14	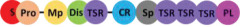	assembly of collagen fibrils myocardial repair **(2)**	
unknown	ADAMTS-6/10	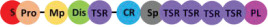	assembly of fibrillin microfibrils (**10**)	WMS **(10)** prolonged QRS **(6)**
unknown	ADAMTS-7/12	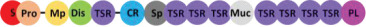	VSMC migration and re-endothelialisation **(7)**	CAD **(7)**
VWF-cleaving protease	ADAMTS-13	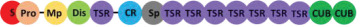	haemostasis	TTP, stroke
unknown	ADAMTS-16/18	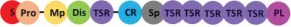	regulation of blood pressure **(16)**	-
unknown	ADAMTS-17/19	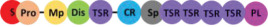	assembly of fibrillin microfibrils **(17)**	WMS **(17)** heart valve disease **(19)**

ADAMTS proteases are generally activated following proteolytic removal of the N-terminal prodomain by subtilisin-type proprotein convertases (e.g. Furin, PCSK6) [14]. With the exception of ADAMTS-13, which circulates in blood, the other ADAMTS family members appear to function in the ECM. The secreted activated enzymes are mainly regulated through inhibition by tissue inhibitors of metalloproteinases (TIMPs) [15] and endocytosis [16].

Distinct ADAMTS subfamilies can be defined on the basis of sequence homology (table 1). They likely evolved through a process of gene duplication resulting in either neo-functionalization or sub-functionalization [17] and some still share a common substrate repertoire.

ADAMTS-1, -4, -5, -8 and -15 are characterized by their ability to cleave PGs and are therefore collectively named ‘proteoglycanases'. Although more distantly related, ADAMTS-9 and -20 also exhibit this distinct proteolytic activity.

ADAMTS-2 is a well-characterized procollagen N-propeptidase that cleaves the N-terminal propeptides of type I, II and III collagen [18]. ADAMTS-3 and ADAMTS-14 show a high degree of homology with ADAMTS-2 and can cleave pro-collagen *in vitro*. ADAMTS-3 is essential for lymphangiogenesis through activation of vascular endothelial growth factor (VEGF)-C [19,20].

The related pairs *ADAMTS7/12*, *ADAMTS6/10*, *ADAMTS16/18* and *ADAMTS17/19* are not well characterised [21]. *ADAMTS10* and *ADAMTS17* mutations give rise to Weill-Marchesani syndrome (WMS)-like spectrum and have been functionally associated with the assembly of fibrillin microfibrils [21]. ADAMTS-6 has also been shown to promote fibrillin-1 microfibril formation [22].

ADAMTS-13, by far the best characterized family member, regulates the function of von Willebrand Factor (VWF) in primary haemostasis [23,24].

Whereas non-cardiovascular roles of the different ADAMTS family members have been discussed in recent excellent reviews [10,14], here, we will discuss in detail the involvement of ADAMTS proteases in cardiovascular biology and disease.

## 2. Proteoglycans and proteoglycanases in cardiovascular physiology and disease

### 2.1. Proteoglycans

Several ADAMTS family members specifically cleave PGs. Because ‘The biology of a protease is really the biology of its substrates' [14], we will briefly discuss the role of PGs in the cardiovascular system. In blood vessels, PGs are mainly expressed by ECs and VSMCs in the tunica intima and media, respectively, where they regulate the biophysical properties of the ECM [3,19]. Moreover, through their interactions with ECM proteins, growth factors and chemokines, PGs regulate a variety of processes such as cell signalling, proliferation, migration and apoptosis [25]. Increased accumulation of PGs is a feature of atherosclerosis [26,27] and aneurysms [28], as well as hereditary diseases such as pediatric aortic valve disease and adult myxomatous mitral valves [29].

PGs are classed according to the predominant GAG covalently attached to serine residues in their protein core, heparin/heparan sulfate (HS) PGs and chondroitin-sulfate/dermatan sulfate (CS/DS) PGs. CS-GAGs contain *D*-glucuronic acid and *N*-acetyl-*D*-galactosamine, whereas in DS-GAGs, the *D*-glucuronic acid is epimerized into *L*-iduronic acid. HS-GAGs contain *D*-glucuronic acid or *L*-iduronic acid alternating with *N*-acetyl-*D*-glucosamine. GAGs are not only important to generate a Donnan osmotic pressure in the vascular tissue owing to their negative charges [4] but also to mediate lipoprotein uptake from the circulation.

Sub-intimal accumulation of lipid-rich and inflammatory deposits (plaques) in medium and large arteries is a hallmark of atherosclerosis (figure 1*a*). The enlargement of plaques hampers the normal blood flow, leading to organ ischemia and tissue necrosis. Plaque rupture with subsequent thrombus formation can cause vascular occlusion leading to potentially fatal cardiovascular events such as myocardial infarction and stroke. PGs such as versican and aggrecan also tend to accumulate in thoracic aortic aneurysms and dissections (TAAD) [28,30,31] and during normal ageing of the aorta [32], resulting in an increased osmotic pressure that is disruptive to the ECM (figure 1*b*). Moreover, PG accumulation can disrupt mechanosensing by VSMCs and create stress-risers in the aortic wall that may predispose to or propagate a dissection [33].

HSPGs such as perlecan and the four transmembrane syndecans are synthesized mainly by ECs in the intima and inhibit pro-atherogenic processes such as lipoprotein retention, infiltration of inflammatory cells, proliferation of VSMCs and thrombosis [34,35]. Since HSPGs are not major ADAMTS substrates, they will not be discussed in detail here. We will instead focus on CSPGs.

CSPGs are mainly expressed by VSMCs in the tunica media [25]. In contrast with HSPGs, CSPGs may initiate atherosclerotic processes by enhancing both deposition and internalization of low-density lipoprotein (LDL) particles that have penetrated into the arterial wall after transcytosis or endothelial dysfunction [27,36] (figure 1*a*). CSPGs can form high-affinity complexes with LDL particles, which are then internalized more efficiently by VSMCs and infiltrated macrophages than native LDLs [37,38]. Once internalized, LDLs enhance intracellular cholesteryl ester synthesis and subsequent foam formation [39]. The interaction between lipoproteins and CSPGs involves an ionic bond between basic amino acids in apoprotein B (apoB) and negatively charged sulfate groups on the GAGs [40]. Moreover, the longer the CS chains, the higher the affinity for LDL [41]. To support the notion that the direct binding of LDL particles to CSPGs is a key step in atherogenesis, transgenic mice expressing LDL particles where positively charged amino acids in apoB had been replaced with neutral ones exhibited significantly less atherosclerotic lesions than mice expressing wild-type apoB [42]. CSPGs comprise large aggregating PGs such as versican and aggrecan and small leucine-rich PGs (SLRPs) such as biglycan and decorin. Owing to their ability to bind both hyaluronan, the only non-sulfated GAG, and lectins, large aggregating PGs are also called hyalectans [43]. Hyalectans have a similar structure, comprising two globular domains at the N- and C-terminus, named G1 and G3, respectively, and a central GAG domain containing attachment sites for GAG chains. The G1 domain binds to hyaluronan, whereas the G3 domain contains the lectin-binding region (figure 1*a*).

Versican is the main hyalectan in the vasculature, where it plays a role in developmental and repair processes such as cell adhesion, proliferation and migration, ECM assembly and inflammation [25,26,44]. It is present in 5 isoforms (V0-V4), generated by alternative splicing within the central GAG-rich region. Expression of versican is essential for normal development of heart and blood vessels [45,46].Versican is involved in various aspects of vascular lesion development and is present in atherosclerotic plaques, restenotic lesions, lesions arising during graft repair and aneurysmal lesions [27].Versican levels increase dramatically in atherosclerosis [27,47], suggesting that its accumulation may in part be responsible for increased LDL deposition/internalization in the vessel wall (figure 1*a*).

Aggrecan, a major CSPG in cartilage, has been recently identified in human aortas [28,30,31,48–53] and, together with versican, it has been shown to accumulate in the aortas of patients with TAAD, resulting in an increased osmotic pressure that is disruptive to the ECM [30] (figure 1*b*). Here, it is important to note that aggrecan has an order of magnitude more CS than versican [54], and therefore more potential to exert osmotic pressure. In addition to CS, aggrecan also contains keratan sulfate (KS) GAGs (where the *D*-galactose replaces hexuronic acid) clustered into a KS-rich region [54].

The protein core of SLRPs such as biglycan is small (40–60 kDa) and characterized by the presence of 11–12 leucine-rich tandem repeats and the attachment of 1–2 CS/DS GAG chains [55]. The leucine-rich tandem repeats bind to collagens, thus regulating collagen fibril formation [56–58].

Biglycan is one of the major PGs found in human atherosclerotic lesions [59–62]. Like aggrecan and versican, biglycan binds to LDL particles, although with reduced affinity owing to the lower number of GAG chains [63]. *Bgn* knockout mice did not show reduced LDL retention in arterial wall, most likely owing to compensation from other CSPGs [64]. However, overexpression of biglycan in mice increased arterial retention of apoB lipoproteins and promoted atherosclerosis [62,65]. Biglycan may also exert an anti-atherosclerotic function. In mice genetically susceptible to develop atherosclerotic lesions (already bearing deletion of either apolipoprotein-E, *ApoE*, or LDL-receptor, *LDLR*), abolishing biglycan expression increased macrophage-mediated plaque inflammation [64]. Biglycan is also considered as an early initiator of aortic stenosis lesion, contributing to wall thickening [66]. Furthermore, biglycan has been implicated in the formation of TAAD, since *Bgn*/*LDLR* double knockout mice showed increased incidence of TAAD [67] and loss of function mutations in *Bgn* results in syndromic early-onset TAAD in humans [68]. *Bgn* knockout mice also showed spontaneous abdominal aortic aneurysms (AAA) [69]. Since biglycan deficiency impairs the formation of collagen fibres in the aortic wall and contributes to the breakdown of elastic fibres [67,70], this will affect the ability of the vessel to sustain tension forces.

### 2.2. Proteoglycanases

Physiological levels of PGs are regulated by the proteoglycanase activity of several ADAMTS family members. ADAMTS-1, -4, -5, -8, -9, -15 and -20 have been shown to cleave, albeit to a different extent, both versican [71–75] and aggrecan [76]. The only ADAMTS cleavage of versican V1 isoform described to date occurs at the Glu441↓442Ala bond within the *β*GAG domain [71–75]. The equivalent cleavage sites in the *α*GAG region in the V0 and V2 isoforms have been identified as Glu1428↓1429Ala [71] and Glu405↓406Gln, respectively [77]. Aggrecan is cleaved by ADAMTS proteases at multiple sites [78], although the cleavage event most detrimental for its function occurs at the Glu392↓Ala393 bond (Uniprot P16112 numbering) in the region between the G1 and G2 domains [79]. Importantly, ADAMTS-mediated cleavage of both aggrecan and versican has been shown to release bioactive fragments. The G1-DPEAAE^441^ versican V1 cleavage fragment, named versikine, is involved in a variety of biological processes such as immune signalling [80] and apoptosis [81], while the G1-NIVSFE^405^ V2 cleavage fragment has been identified as a hyaluronan-binding protein previously identified in human brain [77]. A 32-amino acid long aggrecan fragment generated by MMP-mediated cleavage at Asn360↓Phe361 and ADAMTS-mediated cleavage at Glu392↓Ala393 has been shown to interact with toll-like receptor-2 and excite nociceptive neurons in chondrocytes [82,83]. Following ADAMTS-cleavage at Glu392↓Ala393, the ^393^ARGS neopeptide can diffuse from the ECM into plasma, urine and synovial fluid [84,85]. Among the aforementioned ADAMTS proteases, ADAMTS-4 and -5 show the strongest proteolytic activity against both aggrecan [86,87] and versican [88]; moreover, they have also been shown to cleave the SLRP biglycan at Asn186↓187Cys [86,89]. This proteoglycanase activity exerts important functions in vascular biology and may contribute to cardiovascular diseases such as atherosclerosis and aneurysms, as outlined in the following sections.

#### 2.2.1. ADAMTS-1: friend (in TAAD) or foe (in atherosclerosis)?

ADAMTS-1 was the first member of the family to be described [90]. Early murine knockout models showed the involvement of this enzyme in fertilization and the development of the urogenital system [91–94], but it was soon recognized that ADAMTS-1 plays an important cardiovascular role. The substrate repertoire of ADAMTS-1 appears to extend beyond the CSPGs aggrecan [95] and versican [88]. Reported *in vitro* substrates include nidogen-1 and -2 [96,97], semaphorin 3C [98], Tissue factor pathway inhibitor (TFPI)-2 [99], insulin growth factor binding protein (IGFBP)-2 [100], syndecan-4 [101] and thrombospondins 1 and 2 [98,102]. ADAMTS-1 versicanase activity *in vitro* is considerably weaker than that of either ADAMTS-4 or ADAMTS-5 [88] but appears biologically important in certain developmental processes such folliculogenesis [94]. Mouse studies suggest that ADAMTS-1 versicanase activity within the endocardial cushion contributes to valve maturation and myocardial trabeculation [103,104]. However, ADAMTS-1 proteolysis of non-PG substrates or non-proteolytic activities may be partially responsible for these phenotypes.

In the blood vessels, ADAMTS-1, like ADAMTS-4 and -5, is expressed by ECs, VSMCs and invading macrophages [105–108]. ADAMTS-1 expression was upregulated in human atherosclerotic lesions [105] and its versicanase activity may contribute to plaque instability [71,105]. Transgenic mice overexpressing *Adamts1* on an *ApoE^−/−^* background were investigated by Jönsson-Rylander *et al.* [107], but measurements of atherosclerotic lesion formation were not reported. However, carotid artery ligation in these mice showed a significant increase in neo-intima formation, suggesting an effect of ADAMTS-1 on VSMC migration/proliferation (table 2). In support of this, two miRNAs targeting *ADAMTS1*, miR-265b-3p and miR-362–3p, appeared to inhibit VSMC proliferation and migration [123,124].

**Table 2.** *Adamts* knockout mice and overexpression *in vivo*. AB, aortic banding-induced cardiac hypertrophy; BAV, bicuspid aortic valve; AngII, angiotensin II; PAH, pulmonary arterial hypertension; PPS, pentosan polysulfate; TAAD, thoracic aortic aneurysms and dissections. Genetic manipulation is in mice if not otherwise specified.

enzyme	human disease modelled	intervention	genetic manipulation	effect	reference
ADAMTS-1	arterial injury	carotid artery ligation	transgenic *Adamts1* overexpression *on* Apoe^−/−^ background	enhanced intimal thickening	[107]
	TAAD	AngII	*Adamts1^+/−^*	increased incidence of aneurysm and mortality, hypotension, medial degeneration	[109]
	TAAD	AngII	lentivirus-mediated *Adamts1* knockout	increased incidence of aneurysm and mortality, hypotension, medial degeneration	[109]
ADAMTS-2	cardiac hypertrophy	AB	*Adamts2^−/−^*	increased cardiac hypertrophy, fibrosis and dysfunction	[110]
	cardiac hypertrophy	AB	transgenic *Adamts2* overexpression	decreased cardiac hypertrophy, fibrosis and dysfunction	[110]
ADAMTS-4	atherosclerosis	high fat diet	*Adamts4^−/−^*;* ApoE^−/−^*	increased plaque stability; decreased lipid deposition; decreased macrophage infiltration; decreased versican and aggrecan degradation	[111]
	TAAD	high fat diet/AngII	*Adamts4^−/−^*	reduced incidence of aneurysm and mortality, decreased aortic destruction and versican degradation, decreased macrophage infiltration, decreased VSMC apoptosis	[112]
ADAMTS-5	TAAD	AngII	*Adamts5^−/−^*	reduced blood pressure, increased aortic dilation, accumulation of versican	[50]
	—	—	*Adamts5^−/−^*	aortic and pulmonary valve anomalies, BAV, accumulation of aggrecan and versican	[51,53,113,114]
ADAMTS-6	—	—	*Adamts6^−/−^*	congenital heart defects such as double outlet right ventricle, ventricular hypertrophy, atrial and ventricular septal defects	[115]
ADAMTS-7	atherosclerosis	high fat diet	*Adamts7^−/−^ ApoE^−/−^*	reduced atherosclerotic lesion area	[116]
	vascular injury	wire injury	*Adamts7^−/−^*	reduced neo-intima formation, increased re-endothelialisation	[116,117]
	vascular injury	balloon injury	siRNA-mediated *Adamts7* knockdown in rats	reduced intimal thickening	[118]
	vascular injury	balloon injury	transgenic *Adamts7* overexpression in rats	increased intimal thickening	[118]
ADAMTS-8	PAH	hypoxia-induced PAH	*Adamts8^ΔSM22α^*	decreased right ventricular systolic pressure and right ventricular hypertrophy	[119]
	PAH	hypoxia-induced PAH	*Adamts8^ΔαMHC^*	decreased cardiac hypertrophy, fibrosis and right ventricular dysfunction	[119]
ADAMTS-9			*Adamts9^+/−^*	thickened aortic valve leaflets, myxomatous mitral valves, abnormal myocardial projections and interventricular septae, increased adventitial thickness associated with versican accumulation in the aorta	[120]
ADAMTS-16	hypertension	—	*Adamts16^−/−^* rats	lower systolic blood pressure, decreased arterial stiffness and thickness of the tunica media	[121]
ADAMTS-19	—	—	*Adamts19^−/−^*	aortic regurgitation, aortic stenosis, BAV	[122]

It has also been reported that angiotensin II (Ang II) and other stimuli associated with vascular remodelling induce the expression of ADAMTS-1 in aorta [125]. Since *Adamts1* knockout mice have elevated perinatal mortality [92], heterozygous *Adamts1*^+/−^ mice were tested in a TAAD model [109]. The *Adamts1*^+/−^ genotype exacerbated aortic aneurisms and lethal aortic dissections induced by treatment with the hypertensive factor Ang II. Administration of Ang II induced TAAD in nearly 80% of the *Adamts1*^+/−^ mice and lethal aortic dissections in nearly 50% of these mice, compared with nearly 10 and 8% in wild-type mice [109]. This phenotype resembles that of *Fbn1^C1039G/+^*, a mouse model of Marfan syndrome (MFS) characterized by a low incidence of aortic dissections and ruptures compared to other MFS mouse models [126]. In these ‘Marfan mice’, the introduced *Fbn1* mutation C1039G disrupts the microfibril scaffold, which has complex knock-on effects (including decreased ADAMTS-1 protein levels) owing to the many mechanistic complexities of the fibrillin microfibril niche and its roles in elastic fibre formation, growth factor signalling and VSMC biology [127,128]. However, in human aortic aneurisms, ADAMTS-1 levels are either unchanged or increased [30,129,130].

In conclusion, these findings suggest that ADAMTS-1 may play a detrimental role in the aetiology of atherosclerosis, whereas further studies are required to establish its involvement in the development of aortopathies.

#### 2.2.2. ADAMTS-4, a potential therapeutic target in atherosclerosis and TAAD

ADAMTS-4 cleaves CSPGs such as versican and aggrecan [87,88], brevican [131] and SLRPs such as fibromodulin [86,87] and biglycan [86,89] as well as non-PG substrates such as cartilage oligomeric matrix protein (COMP) [132].

Although ADAMTS-4, like all other ADAMTS family members, with the exception of ADAMTS-13, is predominantly bound to the ECM [86,87], it may diffuse into plasma following cardiovascular damage. Elevated plasma levels of ADAMTS-4 have been consistently found in patients affected by coronary artery disease (CAD) [129,133–135], atherosclerosis [106,136–138] and TAAD [112,129]. Some of these studies associated elevated ADAMTS-4 plasma levels with increased severity of CAD [134,136] and plaque destabilization [137,138]. Increased ADAMTS-4 levels were also found in macrophage-rich areas of human atherosclerotic plaques and unstable coronary plaques [106,138]. Importantly, these findings in humans are in agreement with those obtained from various mouse models (table 2). ADAMTS-4 levels were shown to increase in the atherosclerotic plaques and plasma of *ApoE* knockout [111,138] and *LDLR^−/−^; ApoB^100/100^* [99] double knockout mice as atherosclerosis progressed. Moreover, genetic deletion of *Adamts4* in an *ApoE* knockout background produced a milder atherosclerotic phenotype, with increased plaque stability compared to their littermates [111]. *ApoE*/*Adamts4* double knockout mice also showed reduced cleavage of both versican and aggrecan in arteries, with no compensation shown by other proteoglycanases such as ADAMTS-1 and −5. This was associated with reduced lipid deposition and macrophage infiltration but increased VSMC proliferation [111]. Moreover, in the absence of ADAMTS-4, increased macrophage apoptosis and decreased levels of proinflammatory cytokines were observed [111]. These data suggest that the activity of ADAMTS-4 is associated with more unstable atherosclerotic plaques. Therefore, therapeutic inhibition of ADAMTS-4 may be beneficial at late stages of atherosclerotic development.

Deletion of *Adamts4* was shown to significantly reduce aortic diameter enlargement, aneurysm formation, dissection and aortic rupture in a mouse model of sporadic TAAD induced by a high-fat diet and AgII infusion [112]. These phenotypes were associated with decreased macrophage infiltration, VSMC apoptosis and versican degradation [112]. Expression of *Adamts4* is increased upon Ang II treatment and injection of miR-126a-5p, a miRNA targeting *Adamts4*, has been recently shown to reduce aortic dilation and versican degradation as well as increase survival in these mice [139]. Severe downregulation of this miRNA may be one of the mechanisms responsible for the observed upregulation of *Adamts4* expression in the Ang II model [139].

*In vitro*, *ADAMTS4* knockdown has been shown to reduce macrophage infiltration [129] and VSMC apoptosis [112], two processes that are critical for the development of aortic aneurysms [140], and plaque rapture [141]. In both cases, the versicanase activity of ADAMTS-4 may play a role. Full-length versican is endowed with adhesive properties and its cleavage by ADAMTS proteoglycanases facilitates the migration of immune cells [80,142]. Moreover, versikine, the ADAMTS-generated versican cleavage fragment, can promote apoptosis [81], thus antagonising the anti-apoptotic effect of full-length versican [143,144]. ADAMTS-4 may also be directly involved in apoptosis of VSMCs after translocation to the nucleus and cleavage of poly ADP ribose polymerase-1 (PARP-1), a key molecule in DNA repair and cell survival [112]. Full-length PARP-1 promotes cell survival, whereas cleaved PARP-1 can induce apoptosis [145]. Finally, ADAMTS-4 has been shown to exert pro-apoptotic effects independently of its catalytic activity [146], suggesting that ADAMTS-4 can induce apoptosis through different mechanisms.

Interestingly, ADAMTS-4 expression was increased in the myocardium of rats subjected to hypertension and addition of pentosan polysulfate inhibited both *Adamts4* expression and versican cleavage and ameliorated myocardial function [147].

These clinical observations and *in vivo* data from mouse models of disease point towards multiple roles of ADAMTS-4 in diseased cardiovascular tissues. Whether therapeutic inhibition of ADAMTS-4 could slow the progression of vascular disease in particular warrants further investigation.

#### 2.2.3. ADAMTS-5 regulates cardiovascular proteoglycan levels

ADAMTS-5 has been extensively studied in the context of aggrecan degradation in cartilage and is a validated target for the treatment of osteoarthritis [148]. More recently, a cardiovascular role has emerged for ADAMTS-5, which has been recently reviewed [148] and will be briefly summarized here.

*In vitro*, ADAMTS-5 is a more potent proteoglycanase than ADAMTS-1 and -4 [87,88] and the absence of ADAMTS-5 *in vivo* causes accumulation of cardiovascular PGs (table 2). For example, *Adamts5* knockout mice showed severe anomalies in the pulmonary valve cusps owing to decreased versican cleavage and subsequent versican and aggrecan accumulation [53,113,114]; similarly, they exhibited dilation of thoracic aorta with accumulation of aggrecan and biglycan [51]. It would be interesting to compare this phenotype with that of knock-in mice expressing ADAMTS cleavage-resistant versican (i.e. mutated at the Glu1428↓1429Ala site), called V1R mice, but unfortunately their cardiovascular phenotype has not been described [149]. While the majority of V1R mice were viable and fertile, some of them die of organ haemorrahage after backcrossing [149].

ADAMTS-5 was found to be markedly reduced in aortas of *ApoE* knockout mice, which spontaneously developed atherosclerotic lesions, resulting in accumulation of versican and biglycan [150]. Recombinant ADAMTS-5 reduced the LDL-binding ability of biglycan and released LDL particles from human aortic lesions [150], thus suggesting a role for this enzyme in regulating PG-mediated lipoprotein retention (figure 1*a*). ADAMTS-5 is expressed in both VSMCs [151,152] and macrophages [106], two cell types that also express versican [151,153–155] and TIMP-3 [156–158], the major inhibitor of ADAMTS-4 and -5 [15]. Once a plaque is formed, its stability is associated with high expression levels of TIMP-3 [158,159] and versican [160]. Therefore, atherosclerotic plaque development is impacted by an imbalance between the expression of proteoglycanases, proteoglycanase inhibitors and PGs, where each protein may exert a beneficial/detrimental role at different stages of the process.

In studies of TAAD, the mRNA levels of ADAMTS-5 are found to be decreased [30,161] and this has been recently confirmed at the protein level, both in plasma and in aortic biopsies [152]. Mouse models of TAAD may help to clarify the role of ADAMTS-5 in this disease. In the Ang II model, *Adamts5* knockout mice showed increased aortic dilation, suggesting that ADAMTS-5 plays a non-redundant role in maintaining the viscoelastic properties of aortic ECM [51]. Loss of ADAMTS-5 was associated with increased protein levels of versican and TGF-β [50], a crucial player in the development of TAAD [160]. At the same time, low-density lipoprotein receptor-related protein-1 (LRP-1) expression was downregulated [50], a phenomenon that has itself been shown to exacerbate aortic dilation [162]. Remarkably, in this model, an increase in ADAMTS-1 protein levels did not compensate for the absence of ADAMTS-5, since versican cleavage was severely diminished [50]. This may be explained by the higher intrinsic versicanase activity of ADAMTS-5 [88].

Taken together, these data suggest that the proteolytic activity of ADAMTS-5 is essential in regulating the levels of cardiovascular PGs and that disturbances could affect the disease process in atherosclerosis and TAAD. As a consequence, any treatment for osteoarthritis should aim to spare ADAMTS-5 activity in the blood vessels to avoid imbalance in PG levels [148].

#### 2.2.4. ADAMTS-8, a contributor to pulmonary arterial hypertension

The enzymatic properties of ADAMTS-8, including its substrate repertoire, have not been extensively investigated. The only reported substrate is aggrecan, which was cleaved *in vitro*, but at an extremely high enzyme/substrate ratio [163]. Notwithstanding the homology of ADAMTS-8 with ADAMTS-1, -4 and -5, these data cast doubt on the inclusion of ADAMTS-8 in the proteoglycanase subfamily. *ADAMTS8* has been identified as a tumour suppressor gene in several types of cancers [164] and single nucleotide polymorphisms (SNPs) in the *ADAMTS8* locus have been associated with hypertension in a genome-wide association study (GWAS) [165]. At the protein level, ADAMTS-8 is highly expressed in the lung and the heart [119,166] and, together with ADAMTS-1, -4 and -5, has been detected within human carotid lesions and advanced coronary atherosclerotic plaques [106,107]. ADAMTS-8 expression was increased in the lungs of patients with pulmonary arterial hypertension (PAH) and in mouse/rat models of PAH [119]. In a model of hypoxia-induced PAH, mice bearing a targeted deletion of *Adamts8* in pulmonary arterial smooth muscle cells (*Adamts8^ΔSM22α^*) showed decreased right ventricular systolic pressure and right ventricular hypertrophy compared with wild-type mice, suggesting a crucial role for ADAMTS-8 in the development of PAH [119]. Addition of recombinant ADAMTS-8 to pulmonary artery ECs seemed to exert a pro-inflammatory and pro-apoptotic role [119], similar to its effects on nasopharyngeal carcinoma cell lines [167]. These data suggest potential similarities between the function of ADAMTS-8 in PAH and in cancer. Since ADAMTS-8 is also expressed in the heart, a conditional knockout model with cardiomyocyte-specific deletion of *Adamts8* (*Adamts8^ΔαMHC^*) was generated [119]. These mice showed decreased cardiac hypertrophy, fibrosis and right ventricular dysfunction in response to hypoxia.

Taken together, these results implicate ADAMTS-8 in the development of PAH and potentially other cardiovascular phenotypes, but further studies are necessary to characterize the ADAMTS-8 substrate repertoire, mechanism of action and regulation.

#### 2.2.5. ADAMTS-9 in heart development

Evolutionarily, ADAMTS-9 appears to be the oldest family member, based on its high homology to nematode and fruit fly proteases, named Gon-1 and Adamts-A, respectively [72,168–170]. It is also the largest member of the ADAMTS family, comprising 14 TSR repeats and one Gon-1-like domain at the C-terminus (table 1).

*Adamts9* knockout mice did not survive past 7.5 days of gestation for unknown reasons, but possibly owing to an important role of ADAMTS-9 in the formation and function of primary cilia [171,172]. Heterozygous knockout mice showed a variable penetrance of cardiac anomalies involving the myocardium, mitral valves, aortic valves and proximal aorta, associated with excess versican [120], suggesting that this enzyme is involved in development of the heart (table 2).

In a study of gene expression associated with AAA rupture, aortic tissues from emergency repair of ruptured AAA were compared to tissue from elective surgery. This identified a set of 5 fibroblast-expressed genes exclusively upregulated in AAA, including *ADAMTS9* [173].

## 3. ADAMTS-7 in coronary artery disease

ADAMTS-7 is a potential therapeutic target in atherosclerosis and associated diseases such as CAD. The evidence for a detrimental role has accumulated over the past decade and includes 1) reduced atherosclerosis upon ablation of the *Adamts7* gene in mice (table 2); 2) GWAS that show an association of *ADAMTS7* SNPs with CAD and 3) immunohistochemical detection of ADAMTS-7 in human atherosclerotic plaques.

Knockout of *Adamts7* on an atherosclerotic background reduced total atherosclerotic lesion area in the aorta of *Adamts7*^−/−^/*ApoE*^−/−^ mice by 62% (male) and 54% (female) compared to littermate controls. In *Adamts7*^−/−^/*Ldlr*^−/−^ mice, the reductions were 37% and 52%, respectively [116]. These findings suggest that pharmacological inhibition of ADAMTS-7 activity could slow down the progression of atherosclerosis. *Adamts7*^−/−^ mice also showed an altered response of VSMCs to arterial wire injury [116,117]. They showed greatly reduced neointima formation upon vascular injury, which had been seen previously in rats upon *Adamts7* knockdown with siRNA in a balloon injury model [118]. Using the same model, the opposite effect was seen when ADAMTS-7 was overexpressed [118]. These effects of ADAMTS-7 on VSMC *in vivo* agree with findings *in vitro* [116–118,174].

GWAS have shown that SNPs in the *ADAMTS7* locus are associated with CAD [174]. The SNP that is thought to cause the association is *rs3825807*, of which the G allele is associated with a reduced risk of CAD. It causes a serine to proline substitution in position 214 of the prodomain of ADAMTS-7. The proline is located near recognition motifs of subtilisin-like proprotein convertases such as furin and PCSK6, which activate ADAMTS-7 by proteolytic removal of the inhibitory prodomain. The proline was shown to hamper prodomain removal, which consequently reduces the activation of ADAMTS-7 [174] and potentially mediates the reduced CAD risk associated with the G allele. VSMCs of the G/G genotype for *rs3825807* also migrated less *in vitro* compared to the A/A genotype.

Immunohistochemistry of human atherosclerotic plaques identified ADAMTS-7 protein [174,175]. In carotid plaques, ADAMTS-7 levels were increased in patients with cerebrovascular symptoms compared to patients without these symptoms and high levels also correlated with increased risk of post-operative cardiovascular events [175].

Several *in vitro* substrates of ADAMTS-7 have been reported in the literature, but clear links with atherosclerosis and VSMC behaviour have not been established [117,176,177]. The mass spectrometry-based method terminal amine isotopic labelling of substates (TAILS) was used to identify potential ECM substrates, which identified latent TGF-β binding protein 4 (LTBP4) as a substrate [178]. LTBP4 is a component of microfibrils/elastic fibres in the lung and large blood vessels and is also co-expressed with ADAMTS-7 in the heart [179,180]. It binds to several ECM proteins, including fibrillin-1, fibulin-4 and fibulin-5, which are essential for the formation of elastic fibres in large blood vessels [179,181,182]. Proteolysis of LTBP4 by ADAMTS-7 likely affects this process, but this remains to be investigated *in vivo*. The significance of LTBP4 proteolysis by ADAMTS-7 for atherosclerosis is also currently unclear. However, a recent report showed that the *LTBP4* gene was differentially expressed between plaques from symptomatic and asymptomatic patients [183], suggesting LTBP4 may affect the composition of atherosclerotic plaques.

Whereas several other ADAMTS family members are regulated by the endogenous metalloprotease inhibitor TIMP-3, ADAMTS-7 is more susceptible to inhibition by TIMP-4, which inhibited ADAMTS-7 efficiently at low nanomolar concentrations [178]. Both TIMP-4 and ADAMTS-7 have a restricted tissue distribution with particularly abundant expression in adult cardiovascular tissues [116,184].

In summary, ADAMTS-7 is a potential therapeutic target in CAD and related diseases resulting from atherosclerosis, but more research is needed to validate it as a target and allow a better understanding of the molecular mechanisms involved.

## 4. ADAMTS-13, thrombotic thrombocytopenic purpura and stroke

ADAMTS-13 circulates in blood at a concentration of approximately 6 nM, where it trims newly secreted VWF multimers that would otherwise be too thrombogenic [185] (figure 1*a*). Very low (less than 10%) ADAMTS-13 activity causes the disease TTP and moderately low ADAMTS-13 activity is associated with ischaemic stroke, which is also a feature of TTP. In TTP, the VWF multimers that are too long spontaneously aggregate platelets in the absence of endothelial damage [186]. TTP is a rare, life-threatening condition that most commonly presents in previously healthy young to middle-aged adults, with an annual incidence of 6 per million in the UK [187,188]. They show severe microangiopathic hemolytic anemia (MAHA), severe thrombocytopenia and end-organ damage. The organs most commonly affected are the heart, brain, kidneys and gastrointestinal tract [189]. The MAHA is the result of the microthombi that occlude the small vessels and damage the red blood cells, whereas the severe thrombocytopenia is caused by consumption of the platelets that are caught up in the microthrombi. The end-organ damage results from the occlusion of the small vessels that oxygenate the organs [188]. In a small minority of TTP patients, ADAMTS-13 activity is low because of inherited mutations in the coding region of the *ADAMTS13* gene [189]. The most common form of TTP is immune-mediated TTP (iTTP), involving autoantibodies against ADAMTS-13, which rapidly clear the enzyme from the circulation and inhibit its activity by preventing binding to its substrate VWF [190]. Autoantibodies that target the N-terminal domains up to the Sp domain can be inhibitory, in line with the discovery of several exosites in these domains [190–195]. Treatment of iTTP currently consists of plasma exchange (PEX) to remove pathogenic autoantibodies and provide ADAMTS-13, in conjunction with Rituximab to suppress autoantibody production [186,196]. PEX is critical and lifesaving when patients present at the hospital in an emergency. More recently, PEX is also supplemented with Caplacizumab, a construct consisting of two fused single-domain antibodies that both target the same epitope in the VWF A1 domain and reduce platelet aggregation [197]. Recombinant ADAMTS-13 is currently undergoing a Phase 2 clinical trial, in which it is used to supplement PEX in iTTP patients with the aim to speed up recovery and reduce the use of PEX (ClinicalTrials.gov: NCT03922308). Whereas most antithrombotic agents carry a risk of bleeding, this is unlikely for recombinant ADAMTS-13 because of the unique mechanism by which its activity is regulated. It is not regulated by a physiological inhibitor (e.g. TIMPs, [198]), but by the limited and conditional availability of VWF exosites and scissile bonds. These are normally buried inside the globular VWF A2 domain and only exposed when the A2 domain unfolds upon elevated shear stress in the circulation [199–201]. Ultra low VWF undergoes elevated shear stress when it exits the endothelium and enters the circulation, causing proteolysis by ADAMTS-13, which reduces the size of the multimer and thereby reduces the tensile force exerted upon the molecule, preventing further unfolding and cleavage of VWF A2 domains [199,202]. VWF is the only reported substrate of ADAMTS-13. The specificity of the protease is conferred by exosites in several ADAMTS-13 domains that bind complementary exosites in the VWF A2 domain C-terminal to the scissile bond [193,194,203–205]. In addition, subsites in the Mp domain accommodate VWF side chains either side of the scissile bond and add to the overall specificity of the enzyme [204,206,207].

Whereas ADAMTS-13 activity levels less than 10% can cause TTP, low levels that fall within the normal range (lowest quartile) are associated with increased risk of developing ischemic stroke [208–210]. In patients who present with acute ischemic brain injury, the ratio of VWF antigen levels to ADAMTS-13 activity (VWF:Ag/ADAMTS-13Ac) predicts mortality and is associated with impact on brain function in survivors [211]. Also, in TTP patients who have recovered and are in remission, lower ADAMTS13 activity levels after recovery are associated with stroke [212]. In animal models of ischemic stroke, the administration of recombinant ADAMTS-13 after a stroke appears to be beneficial [213,214].

## 5. Other ADAMTS family members

### 5.1. The procollagenase ADAMTS-2 in myocardial repair

ADAMTS-2 is the major enzyme cleaving the N-propeptide from type I procollagen, thus allowing the assembly of collagen trimers into fibrils/fibres [215]. Although this has primarily been studied in the skin, it may also occur in other tissues. For example, the analysis of hearts from cardiomyopathic patients showed upregulation of *ADAMTS2,* which may reflect the important role of collagen in myocardial repair and scarring. The collagen that is deposited to ‘repair’ myocardial damage requires prodomain removal by ADAMTS-2 for collagen fibres to form. Increased collagen expression is likely to require increased ADAMTS-2 expression. This may also explain the altered *Adamts2* expression in mice treated with isoproterenol, which induces cardiac myopathy and hypertrophy [216] (table 2). In A*damts2* null mice, the detrimental effects of pressure overload on the heart were enhanced [110], possibly owing to disturbed repair mechanisms involving collagen.

### 5.2. ADAMTS-6 in heart development and QRS duration

*ADAMTS6* mRNA has been detected in the mouse heart, specifically in the outflow tract, valves, atria and the ventricular myocardium [115]. The physiological function of ADAMTS-6 is not known but *in vitro* studies suggest that it may be linked to that of fibrillin-1 microfibrils and focal adhesions [22]. Importantly, ADAMTS-6 has been implicated in cardiac biology by a GWAS, which found that two *ADAMTS6* missense variants (S90 L and R603 W) were associated with the duration of the QRS interval of the electrocardiogram [115], which reflects cardiac ventricular depolarization. A prolonged QRS is a predictor of mortality both in the general population and in patients affected by cardiovascular disease [217–219]. The missense variants S90 L in the prodomain and R603 W in the first TSP-1-like domain both severely impair protein secretion [115]. Puzzingly, S90 L was associated with longer QRS and R603 W with shorter QRS duration. In people of European descent, approximately 1/500 is heterozygous for the S90 L variant, whereas R603 W is rare in individuals of European ancestry but is common in people of African decent, where approximately 1/70 is heterozygous (https://gnomad.broadinstitute.org/). Both variants may, however, be pathogenic in a homozygous state and/or function as risk factors for cardiac disease in heterozygous form. A cardiac role for ADAMTS-6 is also in line with the fatal congenital heart defects of mice homozygous for a null mutation in *Adamts6,* which die pre/peri-natally [115]. Their embryonic heart defects comprise the double outlet right ventricle, an atrioventricular septal defect and ventricular hypertrophy. Interestingly, mice hemizygous for the null mutation are viable and do not show structural heart defects but their ventricles express low levels of connexin-43 protein, the main myocardial gap junction protein in mouse and human heart. The reduced levels of connexin-43 appear to have a post-transcriptional cause, as the mRNA levels of the corresponding gene (*Gja1*) were unaffected [115]. In summary, these findings suggest that ADAMTS-6 may play a role in development of the heart and in regulating gap junction-mediated ventricular depolarization.

### 5.3. ADAMTS-10 and cardiovascular manifestations of WMS

Mutations in *ADAMTS10* cause an autosomal recessive form of WMS [220–222]. WMS is a rare inherited disorder of the connective tissues characterized by short stature, brachydactyly, joint stiffness, broad skull, heart defects and a variety of eye abnormalities [222,223]. Three distinct *ADAMTS10* mutations were identified in two consanguineous families and in one sporadic WMS case, including one nonsense mutation and two splice mutations [220]. Among the clinical features of WMS patients bearing *ADAMTS10* mutations were aortic and pulmonary stenosis with dysplastic valves and hypertrophic obstructive cardiomyopathy [220]. Weil-Marchesani syndrome is also caused by mutations in fibrillin-1 (*FBN1*) and *LTBP2*, implicating ADAMTS-10 in fibrillin-1 microfibril biology [21]. Fibrillin-1 microfibrils are ECM assemblies that are essential for the formation of the elastic fibres in blood vessels, lungs, skin, ligaments and other elastic tissues. They also regulate the bioavailability of growth factors of the TGF-β and bone morphogenetic protein (BMP) superfamily [224]. ADAMTS-10 was subsequently confirmed to regulate fibrillin microfibril function, and to bind fibrillin-1 at two sites that coincide with the fibrillin-1 mutations in WMS [225]. It also co-localizes with fibrillin-1 in tissues and accelerates microfibril assembly in fibroblast cultures [226]. Ocular features in WMS caused by *ADAMTS10* mutations may be owing to reduced fibrillin-2 cleavage [227]. Mutations in other genes involved in fibrilin microfibril biology cause the related disorders WMS-like syndrome (*ADAMTS17*) and geleophysic dysplasia (*LTBP3, ADAMTSL2, Fibrillin 1)* [228].

### 5.4. ADAMTS-16, a potential regulator of blood pressure

The function of ADAMTS-16 is currently unknown, with contradictory reports on its possible involvement in male fertility and sex determination [229,230]. So far, the only described substrate is fibronectin [231]. ADAMTS-16 has been identified as a quantitative trait gene (QTG) for blood pressure in humans [232,233] and rats [234]. To investigate the cardiovascular function of ADAMTS-16, *Adamts16* knockout rats have been generated [121] (table 2). These rats show lower systolic blood pressure, decreased arterial stiffness and thickness of the tunica media compared with wild-type rats, suggesting an involvement of ADAMTS-16 in regulating haemodynamics [121]. Moreover, *Adamts16* knockout rats survived longer, although they exhibited renal anomalies [121]. More data are needed to ascertain a possible role of ADAMTS-16 in blood pressure regulation.

### 5.5. ADAMTS-19 in progressive heart valve disease

A recent study of early-onset valvular disease identified mutations in *ADAMTS19* by whole exome sequencing. Four patients in two consanguineous families carried homozygous mutations in *ADAMTS19* causing a large multi exon deletion in one family and a truncated ADAMTS-19 protein in the other family [122]. To confirm causality, *Adamts19* knockout mice were generated (table 2). Of the homozygous *Adamts19* knockout mice, 38% showed aortic valve regurgitation and/or aortic stenosis at three months of age, confirming an important role of ADAMTS-19 in aortic valve physiology. Expression analysis of *lacZ* in *Adamts19* knockout mice showed strong localized expression of *lacZ* by valvular interstitial cells in all four valves around E14.5 and expression by these cells until adulthood. Ultrastructural analysis of the ECM suggested alterations in ECM organization, including PG accumulation. The observation of PG accumulation raises the question whether this is secondary to disturbed valve physiology/cellular function or reduced PG turnover by ADAMTS-19.

## 6. Targeting ADAMTS therapeutically

ADAMTS inhibitors could potentially be used therapeutically to reduce enzyme activity that contributes to or aggravates cardiovascular disease (e.g. for ADAMTS-7). However, it has proven challenging to develop therapeutic metalloprotease inhibitors with sufficient selectivity to prevent side effects caused by cross-inhibition of related metalloproteinases [235]. Selective inhibitors can be either monoclonal antibodies [235] or small molecules, with the latter having the distinct advantage that they can be administered orally. Selective small molecule inhibitors can be designed on the basis of the available tridimensional structure of the target enzyme or identified by high throughput screening (HTS) of large compound libraries. To screen small molecule libraries for their inhibitory potential, purified enzyme and a high throughput activity assay are needed. Typically, high throughput activity assays for proteases involve Förster resonance energy transfer (FRET) technology, where proteolysis of a small peptide generates a fluorescent signal [236]. For ADAMTS-7 we have developed such an activity assay using a small LTBP4 peptide as an efficient substrate and are currently converting this for HTS of small molecule libraries (manuscript in preparation)

On the other hand, where the activity of an ADAMTS family member has proven to be beneficial in a certain pathological context, so-called enhancers or activators may be envisaged. For example, monoclonal antibodies able to increase the catalytic activity of ADAMTS-13 have been reported [237]. Another innovative approach to increase ADAMTS activity may involve interfering with LRP-1-mediated endocytosis. We have recently reported a monoclonal antibody that is able to bind to ADAMTS-5 and block its binding to LRP-1 without interfering with its proteoglycanase activity [238], resulting in accumulation of active ADAMTS-5 in the extracellular milieu. Such an antibody may be used to rescue ADAMTS-5 proteoglycanase activity in mouse models of atherosclerosis and TAAD (table 2).

## 7. Conclusion

ADAMTS family members fulfil multiple distinct roles in cardiovascular tissues (table 1). Several of them are important for cardiac valve embryogenesis and homeostasis (figure 2), but in general the multitude of physiological processes affected by ADAMTS proteases reflects the many functions of the ECM, such as regulating cell behaviour and sequestering of a wide range of cellular growth factors. Much has been learned about ADAMTS proteases from animal studies, but caution should be exercised when extrapolating mouse data to human disease in the absence of clinical studies. In this regard, a conclusion should be supported by high-quality data collected *in vitro, in vivo* and *ex vivo* but so far this goal has been achieved just for few family members. Nevertheless, exciting new findings are published every year, incrementally elucidating the physiological roles of this fascinating family of proteases.

## Data accessibility

This article has no additional data.
